# Plasma cell myeloma initially diagnosed as light-chain deposition disease on liver biopsy: A case report and literature review

**DOI:** 10.1097/MD.0000000000033406

**Published:** 2023-03-31

**Authors:** Ji Yun Jeong, Hyeon Tae Yang, Seung Hyun Cho, Yu Rim Lee, Jinhee Kim, Min Kyu Kang, Jihoon Hong, Joon Ho Moon, An Na Seo

**Affiliations:** a Department of Pathology, School of Medicine, Kyungpook National University, Kyungpook National University Chilgok Hospital, Daegu, South Korea; b Department of Pathology, School of Medicine, Kyungpook National University, Kyungpook National University Hospital, Daegu, South Korea; c Department of Radiology, School of Medicine, Kyungpook National University, Kyungpook National University Chilgok Hospital, Daegu, South Korea; d Department of Internal Medicine, School of Medicine, Kyungpook National University, Kyungpook National University Chilgok Hospital, Daegu, South Korea; e Department of Radiation Oncology, School of Medicine, Kyungpook National University, Kyungpook National University Chilgok Hospital, Daegu, South Korea; f Department of Hematology/Oncology, School of Medicine, Kyungpook National University, Kyungpook National University Chilgok Hospital, Daegu, South Korea.

**Keywords:** cholestatic hepatitis, light-chain deposition disease, liver, plasma cell myeloma

## Abstract

Light-chain deposition disease (LCDD) is a rare condition characterized by the abnormal deposition of monoclonal light chains (LCs) in multiple organs, leading to progressive organ dysfunction. Herein, we report a case of plasma cell myeloma initially diagnosed as LCDD on liver biopsy performed for prominent cholestatic hepatitis.

**Patient concerns:**

A 55-year-old Korean man complained of dyspepsia as the main symptom. On abdominal computed tomography performed at another hospital, the liver showed mildly decreased and heterogeneous attenuation with mild periportal edema. Preliminary liver function tests revealed abnormal results. The patient was treated for an unspecified liver disease; however, his jaundice gradually worsened, prompting him to visit our outpatient hepatology clinic for further evaluation. Magnetic resonance cholangiography revealed liver cirrhosis with severe hepatomegaly of unknown cause. A liver biopsy was performed for the diagnosis. Hematoxylin and eosin staining revealed diffuse extracellular amorphous deposits in perisinusoidal spaces with compressed hepatocytes. The deposits, which morphologically resembled amyloids, were not stained by Congo red but stained strongly positive for kappa LCs and weakly positive for lambda LCs.

**Diagnoses:**

Therefore, the patient was diagnosed with LCDD. Further systemic examination revealed a plasma cell myeloma.

**Interventions:**

Fluorescence in situ hybridization, cytogenetics, and next-generation sequencing tested in bone marrow showed no abnormalities. The patient initially received bortezomib/lenalidomide/dexamethasone as the treatment regimen for plasma cell myeloma.

**Outcomes:**

However, he died shortly thereafter because of coronavirus disease 2019 complications.

**Lessons:**

This case demonstrates that LCDD may present with sudden cholestatic hepatitis and hepatomegaly, and may be fatal if patients do not receive appropriate and timely treatment because of delayed diagnosis. Liver biopsy is useful for the diagnosis of patients with liver disease of unknown etiology.

## 1. Introduction

Light-chain deposition disease (LCDD) is a rare condition (monoclonal gammopathy) characterized by the abnormal deposition of light chains (LCs) in multiple organs, leading to progressive solid organ dysfunction. The kidneys are the most commonly affected organs and are responsible for early clinical symptoms. Recently, the International Kidney and Monoclonal Gammopathy Research Group Consensus described LCDD as part of monoclonal gammopathy of renal significance, a widespread B-cell proliferative disorder that leads to the production of monoclonal immunoglobulins that are toxic to the kidneys.^[1,2]^

LCDD is categorized as a monoclonal immunoglobulin deposition disease (MIDD) in the World Health Organization classification of plasma cell disorders.^[1]^ The pathophysiology underlying LCDD involves end-organ damage associated with abnormal deposition of LCs produced by plasma cells, regardless of the plasma cell burden or LC production level.^[1]^ The liver is the second most commonly affected organ after the kidneys, but liver involvement rarely leads to clinically significant disease. Herein, we present the case of a patient who was initially diagnosed with plasma cell myeloma (PCM) after visiting a hepatology clinic for dyspepsia and jaundice due to marked liver involvement in LCDD.

## 2. Case report

This study was approved by the Institutional Review Board of Kyungpook National University Chilgok Hospital (No.2023-01-029). The requirement for informed consent was waived because the patients’ personal information was anonymized.

**Figure 1.**
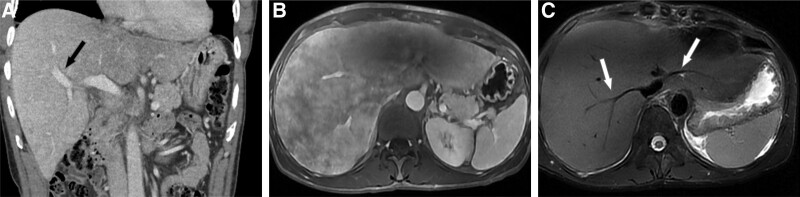
(A) Coronal computed tomography obtained during the portal venous phase shows periportal edema (black arrow) and mildly heterogeneous, mottled parenchymal enhancement in the liver. The volume of the left hepatic lobe is decreased and the volume of the right hepatic lobe is increased. (B) After 3 months, contrast-enhanced T1-weighted axial magnetic resonance (MR) obtained during the portal venous phase reveals more extensively heterogeneous, mottled parenchymal enhancement in the liver. The hypotrophy of the left liver and hypertrophy of the right liver is still noted. In addition, the surface contour of the liver is slightly lobulated. (C) Respiratory-triggered fat-suppressed T2-weighted image shows decreased hepatic vein diameter (white arrows). These MR findings suggest chronic liver disease.

**Figure 2.**
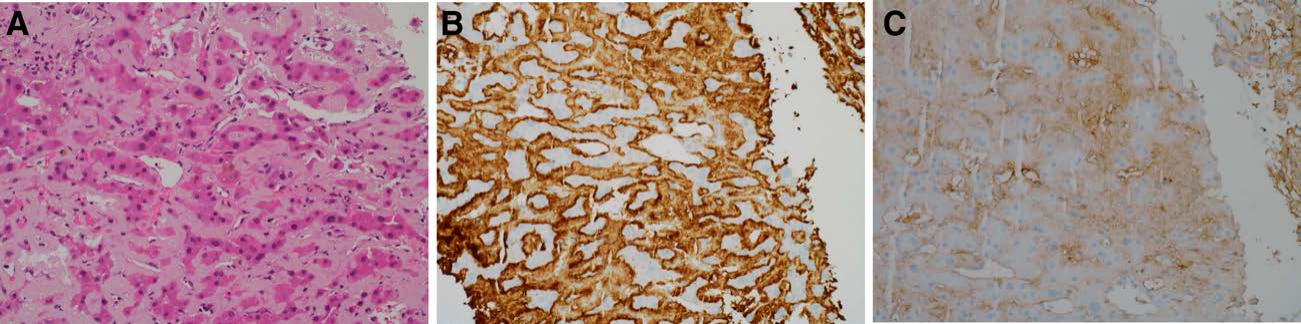
(A) Hematoxylin and eosin staining in the liver biopsy specimen show deposition of amorphous and eosinophilic materials in the perisinusoidal area (20 × objective). On Congo red staining, no green birefringence was noted under a polarized light microscope. (B) Immunohistochemical staining for kappa light chains showed strong and diffuse expression, (C) but lambda light chains showed focal and weak expression (20 × objective).

**Figure 3.**
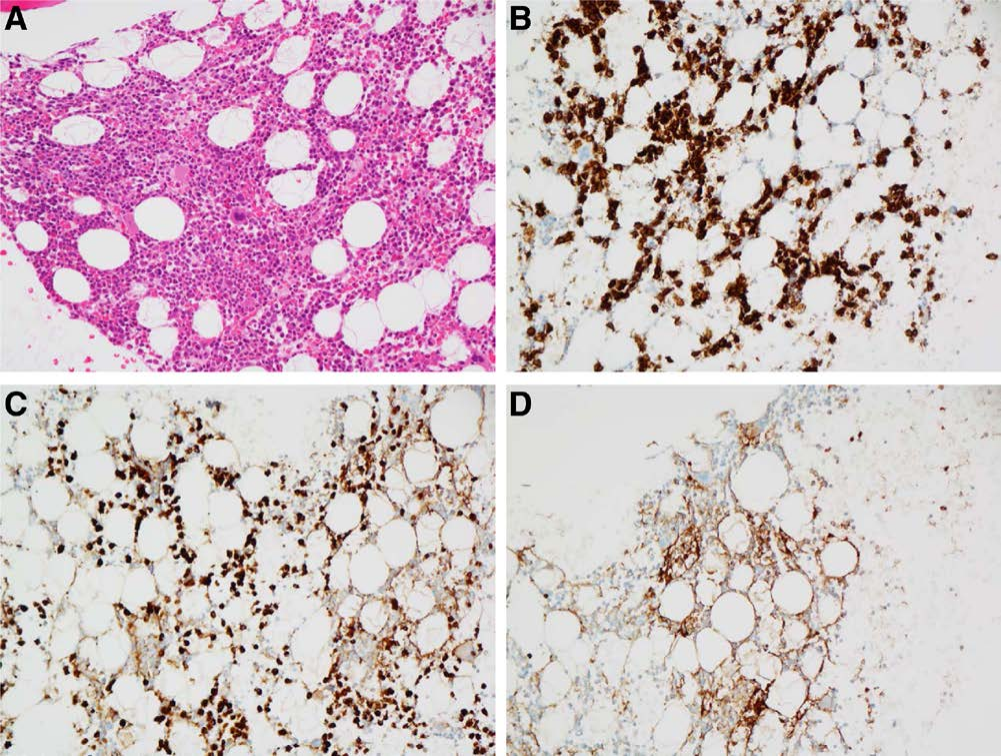
(A) Hematoxylin and eosin staining in the bone marrow specimen shows an increased proportion of neoplastic plasma cells (20 × objective). (B) These neoplastic plasma cells were strongly expressed on immunohistochemical staining for CD138 (20 × objective). (C) These cells also showed strong and diffuse expression on immunohistochemical staining for kappa light chains, (D) but showed focal expression on immunohistochemical staining for lambda light chains (20 × objective).

In August 2022, a 55-year-old Korean man visited an outside hospital because of dyspepsia for 1 month and weight loss of 10 kg in 1 year. Initial abdominal computed tomography revealed mildly decreased and heterogeneous attenuation in the liver with mild periportal edema (Fig. 1A). A calcified nodule was also observed in the posterior segment of the liver. In October 2022, preliminary laboratory tests performed at another hospital revealed mild anemia (red blood cell count, 3.90 × 10^6^/µL; hemoglobin level, 12.7 g/dL; and hematocrit level, 36.0%) and erythrocyte sedimentation rate of 30 mm. Preliminary liver function test revealed significantly abnormal levels: aspartate aminotransferase, 55.5 U/L; alanine aminotransferase, 24.3 U/L; alkaline phosphatase, 1532 U/L; total bilirubin, 1.67 mg/dL; direct bilirubin, 0.91 mg/dL; gamma-glutamyl transferase, >1500 U/L; and prothrombin time international normalized ratio, 12.3. The patient was treated for an unspecified liver disease; however, his jaundice gradually worsened. Therefore, he visited our outpatient hepatology clinic in December 2022 for further evaluation. He had no significant family or past histological findings. Although he had lost 10 kg in 1 year, other significant symptoms had not appeared until recently. He denied any history of smoking but regularly drank 10 single shots (50 mL) of Soju (popular alcoholic beverage in Korea) (20% alcohol by volume) over 20 years. To improve his symptoms, he had been taking herbal medicine for the past 2 months. Preliminary liver function tests performed 3 months after the onset of symptoms showed more severe abnormal levels: aspartate aminotransferase, 110 U/L; alanine aminotransferase, 57 U/L; alkaline phosphatase, 794 U/L; total bilirubin, 4.21 mg/dL; direct bilirubin, 2.57 mg/dL; gamma-glutamyl transferase, 2846 U/L; and prothrombin time-international normalized ratio, 13. Serological tests for hepatitis B virus, hepatitis C virus, and autoimmune hepatitis were negative. Moreover, no signs of mild anemia were observed (red blood cell count, 3.98 × 10^6^/µL; hemoglobin concentration level, 13.1 g/dL; and hematocrit level, 39.1%). A tumor marker level test revealed significantly elevated levels of carbohydrate antigen 19-9 (1572.30 U/mL) and β2-microglobulin (3.91 mg/L). Urinalysis revealed dark yellow urine, and bilirubin (3+) was detected. Magnetic resonance cholangiography revealed liver cirrhosis with severe hepatomegaly, but no evidence of splenomegaly or biliary dilatation was noted (Fig. 1B and C). Mild ascites in the pelvic cavity were also observed. However, no abnormalities were observed in either of the kidneys. For diagnosis, an ultrasound-guided needle biopsy of the liver was performed using an 18-gauge TSK gun. Hematoxylin and eosin staining of the liver biopsy specimen showed diffuse extracellular, pink-colored amorphous deposits in the perisinusoidal spaces, and most of the hepatocytes were compressed by the deposits (Fig. 2A). To differentiate it from amyloidosis, Congo red staining was performed, but no apple green birefringence was noted. Immunohistochemically, these deposits were strongly positive for kappa LCs (Fig. 2B) and weakly positive for lambda LCs (Fig. 2C). Accordingly, the patient was diagnosed with LCDD and further systemic examination was performed. ^18^F-fluorodeoxyglucose positron emission tomography revealed no significant abnormal uptake (maximum standardized uptake value of 2.8), with large amounts of ascites and edema in the lower trunk and extremities. However, the serum immunofixation test indicated a distinct M-spike in the β-region, and a monoclonal gammopathy pattern was identified. In contrast, no monoclonal gammopathy pattern was observed on the urine immunofixation test. Bone marrow aspiration and trephine biopsy revealed an increased proportion of neoplastic plasma cells (22.6%) (Fig. 3A–D). Therefore, the patient was diagnosed with PCM. In cytogenetic analysis, bone marrow Karyotype with 46, XY [20] was observed, and no numerical or structural abnormalities of chromosomes were found. In addition, no variation in Tier 1 to 3 was observed in the performance of next-generation sequencing using customized multiple myeloma panels consisting of 24 genes via the MiSeqDx instrument (Illumina, CA). In fluorescence in situ hybridization, *CKS1B*/*CDKN2C* (P18) amplification/deletion, *IGH*/*MAFB* rearrangement, *IGH*/*MAF* rearrangement, *IGH*/*FGFR3* rearrangement, *IGH*/*CCND1* rearrangement, *TP53* deletion, and atypical anomaly were also not observed. Serum and urine protein electrophoresis tests revealed that the level of free kappa LCs significantly increased (4296.0 mg/L), whereas that of free lambda LCs slightly elevated to 35.44 mg/L. These findings are compatible with those of LCDD. Transthoracic echocardiography revealed septal hypertrophy and no abnormalities in left ventricular systolic function. The patient received bortezomib/lenalidomide/dexamethasone (1 cycle) as the treatment regimen for PCM. After 3 days, the patient tested positive for coronavirus disease 2019 and suffered multiple infectious complications, such as general weakness, seizures, and dyspnea, in January 2023, and died 1 month after the liver biopsy.

## 3. Discussion

LCDD is a rare systemic disease characterized by tissue deposition of abnormal monoclonal LCs due to plasma cell dyscrasia.^[3,4]^ As this disorder was first described in 1976 by Randall et al^[3]^ in 2 patients with end-stage renal disease showing granular deposition of free LCs,^[5]^ the kidneys are usually the most commonly affected organ and are responsible for early clinical manifestations, ranging from asymptomatic proteinuria to nephrotic syndrome or renal insufficiency.^[4,6–8]^ Notably, extrarenal involvement appears to be more common in patients with PCM. Among other extrarenal organs, the liver is the second most commonly affected organ after the kidneys.^[4,9]^ Because liver involvement in LCDD is commonly associated with kidney lesions, cases of liver involvement without kidney involvement are extremely rare.^[10]^ In the present case, cardiac involvement was noted after worsening symptoms, but no kidney involvement was observed, and the clinical signs were dominated by hepatic manifestations, which led to more diverse differential diagnoses. Our patient presented with massive hepatomegaly without splenomegaly, severe cholestasis, markedly elevated liver function biomarker levels, and normal renal function. Liver biopsy showed diffuse extracellular, pink-colored amorphous deposits in the perisinusoidal spaces, with a predominance of kappa isotypes, and the hepatocytes were compressed by the perisinusoidal deposits. When this morphologic finding of extensive homogenous eosinophilic deposits is observed, amyloidosis or amyloid-like fibronectin deposits should be included in the differential diagnosis.^[8,10,11]^

Liver involvement in LCDD tends to be present along the sinusoids or perisinusoidal space, as well as in the portal areas along the basement membranes of the biliary tracts, and the liver parenchyma is usually preserved.^[4,10,12]^ Unlike amyloid LC amyloidosis, disorganized, electron-dense, and granular deposits are characteristic features of LCDD.^[4,13]^ In addition, LC deposits in LCDD are not stained by Congo red and do not exhibit green birefringence under polarization.^[5,9]^ Notably, diseases in which extracellular deposits show negative Congo red staining indicate Waldenstrom macroglobulinemia and amyloid-like fibronectin in addition to LCDD.^[11,14]^ For differential diagnosis, clinical correlation, and mass spectrometric analysis may be helpful.^[11]^ Amyloid-like fibronectin deposits in the liver were first described in 2021 by Yasir et al^[11]^ in a patient with hepatocellular carcinoma and a patient with non-neoplastic liver. They suggested that fibronectin is produced and secreted by hepatocytes and exists in both tissue and plasma forms.^[11]^ They proposed 2 hypotheses for fibronectin deposits: plasma fibronectin polymerizes to form β-pleated sheets with amyloid-like features under laboratory conditions^[11,15]^ and similar amyloid-like aggregation of fibronectin is formed in tissues under a stressed cellular microenvironment.^[11,16]^ Notably, after searching large-scale databases, fibronectin deposition was found to be extremely rare in both hepatocellular carcinoma and general surgical liver specimens.^[16]^

LCDD is usually diagnosed in patients who are in their mid-50s or -60s (20–91 years), with a male predominance (approximately 2.5-fold).^[5,9,17]^ PCM accounts for 20 to 30% of MIDD cases, whereas monoclonal gammopathy of renal significance accounts for 65 to 75% of MIDD cases.^[18]^ LC deposits in LCDD mostly show the kappa isotype (up to 80%), whereas those in amyloid light-chain amyloidosis indicate lambda isotype.^[8,19]^ The primary defect involves multiple mutations in the immunoglobulin LC-variable region, with kappa LCs of VκIV type being particularly overrepresented.^[6,20]^ However, lambda LC deposition was observed in 15 to 20% of cases, and LC and heavy chain depositions were observed in <10% of cases.^[5]^ Our patient was a 55-year-old man who initially visited the hepatology clinic of our hospital presenting with symptomatic hepatic manifestations and was diagnosed with PCM based on further laboratory findings after assessing LCDD via liver biopsy. Although he lost 10 kg in 1 year, no other symptoms, particularly bone pain in the PCM, were reported before the appearance of dyspepsia, and jaundice was the chief complaint. Intrahepatic cholestasis may result from cytolysis and compression of the bile ducts by portal vein deposits of LCDD.^[9,10]^

As the clinical manifestations of LCDD are dependent on the degree and nature of the organs involved, variable chain deposition does not appear to influence the clinical course.^[4,5]^ Furthermore, older age, PCM, and extrarenal LCD are associated with a high risk and poor prognosis.^[17,21]^ The median overall survival in patients with LCDD varies from 4 to 14 years.^[17,22]^

Unfortunately, owing to the rarity of the disease and lack of randomized clinical trials, there is currently no universally accepted standard treatment option for LCDD.^[1]^ Instead, the main goal of LCDD treatment is to suppress LC production and organ function impairment.^[1]^ The treatment plan is similar to that of PCM, primarily involving proteasome inhibitors (bortezomib), immunomodulatory agents (lenalidomide and thalidomide), and autologous stem cell transplantation.^[1]^ Taken together, large-scale multicenter studies are warranted to clarify the effect of various chemotherapies and autologous stem cell transplantation on patients with LCDD.

In summary, we describe a case of PCM initially diagnosed as LCDD based on liver biopsy performed for prominent cholestatic hepatitis. This case demonstrates that LCDD may present with sudden cholestatic hepatitis and hepatomegaly, and may be fatal if patients do not receive appropriate and timely treatment because of delayed diagnosis. We first diagnosed LCDD via a liver biopsy, and after further examination, the patient was diagnosed with PCM. Liver biopsy is useful for the diagnosis of patients with liver disease of unknown etiology.

## Author contributions

**Conceptualization:** An Na Seo.

**Data curation:** Ji Yun Jeong, Hyeon Tae Yang, Seung Hyun Cho, Yu Rim Lee, Jinhee Kim, Min Kyu Kang, Jihoon Hong, Joon Ho Moon, An Na Seo.

**Formal analysis:** Ji Yun Jeong, An Na Seo.

**Funding acquisition:** An Na Seo.

**Investigation:** Hyeon Tae Yang, An Na Seo.

**Methodology:** Ji Yun Jeong, Hyeon Tae Yang, Seung Hyun Cho, Yu Rim Lee, Jinhee Kim, Min Kyu Kang, Jihoon Hong, Joon Ho Moon, An Na Seo.

**Project administration:** An Na Seo.

**Writing – original draft:** Ji Yun Jeong, An Na Seo.

**Writing – review & editing:** Ji Yun Jeong, Hyeon Tae Yang, Seung Hyun Cho, Yu Rim Lee, Jinhee Kim, Min Kyu Kang, Jihoon Hong, Joon Ho Moon, An Na Seo.
